# Improved coronary artery interpretability without heart rate control for Coronary CT Angiography (CCTA) performed on a dedicated cardiac CT scanner using a second-generation motion correction algorithm

**DOI:** 10.1016/j.ahjo.2025.100566

**Published:** 2025-06-25

**Authors:** David Playford, Tom Phillips, Enayet Chowdhury, Bryan Wai, Daneh Turner, Leighton Kearney

**Affiliations:** a School of Medicine, The University of Notre Dame, Fremantle, Australia; b Advara HeartCare, Leabrook, Adelaide, Australia; c GE Healthcare, 8-241 O'Riordan street, Mascot, NSW 2020, Australia

To The Editor

Traditional Coronary Computed Tomography Angiography (CCTA) requires heart rate (HR) control, usually with the use of pre-procedural beta blockade, in order to minimise cardiac motion artifact that may interfere with image quality. New software algorithms applied to post-processing of images may allow for improvements in coronary lumen delineation at a faster heart rate than had been previously possible.

We undertook a randomised controlled blinded research study to assess image quality of CCTA data processed by a new, second-generation motion correction algorithm compared to standard algorithms in 3 cohorts with distinct heart rate (HR) ranges. The current report contains methods and findings from the review of the assessed images.

We included 75 patients who underwent CCTA performed on a modern CT scanner (CardioGraphe, GE HealthCare) in three separate clinical sites in Australia. The inclusion criteria involved 3 equal cohorts based on heart rate following matching by age and gender: Cohort 1, HR <65 beats per minute (bpm) (*n* = 25); Cohort 2, HR 65–75 bpm (n = 25), and; Cohort 3, HR 76–90 bpm (n = 25). We excluded patients with prior coronary artery bypass surgery, intracardiac devices, prior surgical or transcatheter valve intervention, non-sinus rhythm, poor image quality due to factors other than coronary motion (such as excessive noise, poor contrast timing or coronary artery contrast opacification) and inappropriate cardiac cycle phase of acquisition. An appropriate cardiac cycle phase of acquisition was defined according to the following criteria: Mid-diastolic phase for HR ≤ 70 beats per minute (bpm); Either mid-diastolic or end-systolic phase for HR 71 to 74 bpm, and: End-systolic phase for HR ≥ 75 bpm.

These 75 de-identified CCTA scans (25 in Cohort 1 at HR < 65 bpm, 25 in Cohort 2 at HR 65–75, and 25 in Cohort 3 at HR 76–90) underwent both motion correction (SnapShot Freeze 2, SSF2, GE HealthCare) and the standard algorithm, thus creating a total of 6 distinct groups. All scans were then randomised and the axial images were provided to two independent CCTA cardiologists, blinded to the heart rate or motion correction status of each patient. Each cardiologist then graded image the quality over 18 segments (1 = worst, 4 = best, N/A = not assessable). An independent research panel performed unblinding and reader scores were averaged for analysis, with any segments “N/A” being excluded. Both standard and motion correction scores were compared across each HR group, and then stratified by heart rate using Wilcoxon matched pairs signed-ranks testing. An interpretable coronary segment was defined as average image quality score between 2 and 4, and a non-interpretable segment defined as an average score < 2. Overall interpretability was calculated as the percentage of interpretable segments divided by the total number of evaluable segments. Interpretability was further classified per-artery and per-patient for both the standard and motion-correction algorithm. Pair-wise McNemar or Fisher's exact test was used to compare the difference in interpretability for any given pair of studied images between each algorithm. All statistical analysis was performed using STATA version 18.0, using by Wilcoxon matched pairs signed-ranks test on a per-segment, per-artery and per-patient basis.

The 75 included patients were 56.8 % male, with a mean age of 63.6 ± 11.8 yrs., BMI 28.9 ± 11.8 kg/m^2^ (obesity defined as BMI ≥ 30 kg/m^2^ in 36.5 %), summarised in Table 1. The median coronary artery calcium score was 18, and interquartile range was 0 to 280. The two cardiologists, blinded to the heart rate or motion correction status of each image, reported a total of 960 (standard) and 972 (motion correction) coronary segments. 390 segments were not evaluable either because of anatomical variants or the lumen could not be adequately visualised (due to small vessel calibre), leaving 601 image segment pairs for 73 patients available for analysis. Overall, the mean image quality score and interpretability were 2.8 ± 0.8 and 88.9 %, 3.1 ± 0.5 and 98.7 %, using the standard and motion-correction algorithms on per-segment level, respectively (*p* < 0.001). A higher heart rate favoured the motion correction algorithm vs the standard algorithm (p = ns for HR < 65, p < 0.001 for HR ≥ 65 and HR > 75), particularly RCA interpretation – summarised in Table 2. Using the standard algorithm, we found a progressive and significant decline in reported image quality with higher heart rates, with only the slowest heart rate (<65 bpm) consistently associated with interpretability above 90 % (see Table 2). In contrast, we observed a small deterioration in image quality at the highest heart rates (≥75 bpm) with the motion correction algorithm, but otherwise consistently high interpretability.

**Table 1:** Demographic and calcium score information for the study cohort. Cohort 1 is restricted to heart rate (HR) <65 beats per minute (bpm), Cohort 2 to HR between 65 and 74 bpm, and cohort 3 ≥ 75 bpm. Age and body mass index (BMI) are presented as mean ± standard deviation (SD), sex as percentage of males, with the number against the total number in brackets, and the coronary artery calcium (CAC) score as the absolute Agatston score as median, followed by the interquartile range (IQR).

	Total (*n* = 75)	Cohort 1 (HR <65, n = 25)	Cohort 2 (HR 65–74, *n* = 25)	Cohort 3 (HR ≥75, n = 25)	Overall *p*-value	Cohort 1 vs 2 p-value	Cohort 1 vs 3 p-value	Cohort 2 vs 3 p-value
Age (yrs, ±SD)	63.7 (11.8)	65.9 (7.1)	59.3 (14.4)	66.0 (11.8)	0.07	0.046	0.98	0.08
BMI (kg/m^2^, ±SD)	28.8 (5.3)	28.3 (6.1)	28.5 (5.5)	29.7 (4.1)	0.61	0.93	0.36	0.39
Male, % (n/total)	56 % (42/75)	56 % (14/25)	40 % (10/25)	72 % (18/25)	0.07	0.26	0.24	**0.02**
CAC score (median, IQR)	17.0 (0.0–255.0)	1.5 (0.0–87.5)	1.0 (0.0–73.0)	217.0 (6.0–531.5)	**0.006**	0.95	**0.01**	**0.004**

**Table 2:** Image quality scores of segment, artery, and interpretability among the two reconstruction algorithms. SD = Standard Deviation. Overall interpretability Score and Interpretability: The first bracketed number refers to the number of segments, arteries or patients within the category, and the second bracketed number refers to the total number of segments being considered. * p-value <0.05 considered significant ** per-artery refers to the interpretability overall for the RCA, left main, LAD and Circumflex. **Per-patient refers to the interpretability overall for that individual.

	Overall (n = 75)			Cohort 1 (heart rate < 65 bpm, n = 25)			Cohort 2 (heart rate 65–74 bpm, n = 25)			Cohort 3 (heart rate ≥ 75 bpm, *n* = 25)		
	Standard	Motion correction	p-Value*	Standard	Motion correction	p-Value*	Standard	Motion correction	p-Value*	Standard	Motion correction	p-Value*
Overall score (±SD)	2.8 (0.8)	3.1 (0.5)	<0.001	3.2 (0.5)	3.2 (0.4)	0.87	2.8 (0.7)	3.2 (0.4)	<0.001	2.3 (0.8)	3.0 (0.6)	<0.001

Overall interpretability score (average for all readers)												
Score 1- < 2	11.1 % (67/601)	1.3 % (8/601)	<0.001	1.5 % (3/200)	0.5 % (1/200)	0.63	6.8 % (14/206)	0.5 % (1/206)	<0.001	25.6 % (50/195)	3.1 % (6/195)	<0.001
Score 2- < 3	28.6 % (172/601)	14.0 % (84/601)	<0.001	16.5 % (33/200)	7.5 % (15/200)	0.01	29.6 % (61/206)	8.7 % (18/206)	<0.001	40.0 % (78/195)	26.2 % (51/195)	0.005
Score 3- < 4	54.4 % (327/601)	76.4 % (459/601)	<0.001	71.0 % (142/200)	85.5 % (171/200)	<0.001	59.7 % (123/206)	78.2 % (161/206)	<0.001	31.8 % (62/195)	65.1 % (127/195)	<0.001
Score 4	5.8 % (35/601)	8.3 % (50/601)	0.051	11.0 % (22/200)	6.5 % (13/200)	0.08	3.9 % (8/206)	12.6 % (26/206)	<0.001	2.6 % (5/195)	5.6 % (11/195)	0.15

Artery mean score (all segments within each artery)												
RCA (±SD)	2.6 (0.9)	3.3 (0.5)	<0.001	3.2 (0.6)	3.3 (0.4)	0.04	2.7 (0.7)	3.4 (0.4)	<0.001	2.0 (0.8)	3.1 (0.6)	<0.001
LM (±SD)	3.3 (0.5)	3.5 (0.4)	0.003	3.4 (0.3)	3.4 (0.3)	1.00	3.4 (0.4)	3.6 (0.3)	0.06	3.1 (0.6)	3.4 (0.4)	0.004
LAD (±SD)	2.8 (0.7)	2.9 (0.5)	<0.001	3.1 (0.5)	3.0 (0.4)	0.66	2.8 (0.7)	3.0 (0.5)	0.002	2.3 (0.7)	2.7 (0.5)	<0.001
LCX (±SD)	2.9 (0.7)	3.1 (0.4)	0.01	3.4 (0.4)	3.2 (0.3)	0.052	2.8 (0.5)	3.1 (0.3)	0.007	2.5 (0.7)	3.0 (0.5)	0.001

Interpretability (per-segment, per-artery and per-patient)												
Per-segment	88.9 % (534/601)	98.7 % (593/601)	<0.001	98.5 % (197/200)	99.5 % (199/200)	0.62	93.2 % (192/206)	99.5 % (205/206)	<0.001	74.4 % (145/195)	96.9 % (189/195)	<0.001
Per-artery**	85.9 % (249/290)	97.2 % (282/290)	<0.001	97.9 % (94/96)	99.0 % (95/96)	1.00	90.9 % (90/99)	99.0 % (98/99)	0.008	68.4 % (65/95)	93.7 % (89/95)	<0.001
Per-patient**	61.6 % (45/73)	90.4 % (66/73)	<0.001	91.7 % (22/24)	95.8 % (23/24)	1.00	72.0 % (18/25)	96.0 % (24/25)	0.03	20.8 % (5/24)	79.2 % (19/24)	<0.001

In summary, in this randomised, blinded expert analysis of the use of new motion correction software during post-processing of CCTA studies, we demonstrated excellent interpretability of most coronary segments at heart rates up to 90 bpm, potentially decreasing beta blocker requirements, and improving waiting times and CTCA efficiency.

## CRediT authorship contribution statement

**David Playford:** Conceptualization, Data curation, Investigation, Methodology, Writing – original draft, Writing – review & editing. **Tom Phillips:** Conceptualization, Funding acquisition, Writing – original draft, Writing – review & editing. **Enayet Chowdhury:** Data curation, Formal analysis, Investigation, Methodology, Software, Writing – original draft, Writing – review & editing. **Bryan Wai:** Investigation, Writing – original draft, Writing – review & editing. **Daneh Turner:** Conceptualization, Funding acquisition, Writing – review & editing. **Leighton Kearney:** Conceptualization, Data curation, Formal analysis, Funding acquisition, Methodology, Project administration, Resources, Supervision, Writing – original draft, Writing – review & editing.

## Declaration of competing interest

David Playford, NONE.

Tom Phillips, NONE.

Enayet Chowdhury NONE.

Bryan Wai, NONE.

Daneh Turner, Employee GE HealthCare. Disclosure is mitigated as follows: This author had no role in the design of the study, the data collection, or the interpretation of the results.

Leighton Kearney, NONE.

